# Birthweight and Breastfeeding Shape Patterns of Biological Aging Across Adult Organs

**DOI:** 10.1111/acel.70762

**Published:** 2026-10-09

**Authors:** Wenming Wei, Xin Qi, Bolun Cheng, Boyue Zhao, Dan He, Jingni Hui, Jin Feng, Shiqiang Cheng, Xuena Yang, Chuyu Pan, Yifan Gou, Yan Wen, Huan Liu, Yumeng Jia, Li Liu, Feng Zhang

**Affiliations:** ^1^ NHC Key Laboratory of Environment and Endemic Diseases, School of Public Health, Health Science Center Xi'an Jiaotong University Xi'an Shaanxi China; ^2^ Collaborative Innovation Center of Endemic Diseases and Health Promotion in Silk Road Region Xi'an Shaanxi China; ^3^ Precision Medicine Center The First Affiliated Hospital of Xi'an Jiaotong University Xi'an P. R. China

**Keywords:** allostatic load, biological aging, birthweight, breastfeeding, early‐life nutrition, organ‐specific aging

## Abstract

Early‐life nutrition may influence long‐term health, but its impact on organ‐specific biological aging is unclear. We investigated associations of birthweight and breastfeeding with multi‐organ aging and potential mediating pathways in adulthood. Using UK Biobank data, we examined associations of retrospectively reported birthweight (*n* = 21,446) and breastfeeding history (*n* = 29,713) with 19 proteomics‐derived aging indices, comprising 18 organ‐specific indices and one composite index. Multivariable linear regression assessed individual and joint associations, effect modification by sex and socioeconomic factors, and mediation by allostatic load (AL) and C‐reactive protein (CRP). Higher birthweight was inversely associated with aging across 11 organ systems (all FDR‐adjusted *p* < 0.05), with strongest associations in the intestine (*β* = −0.069), esophagus (*β* = −0.055), and stomach (*β* = −0.053). Breastfeeding exposure was associated with slower aging of the intestine (*β* = −0.047) and adrenal gland (*β* = −0.030), but modestly faster thyroid aging (*β* = 0.033). The combination of higher birthweight and breastfeeding was associated with favorable aging profiles, particularly in the intestine (β = −0.13), stomach (β = −0.09), and kidney (β = −0.08). Associations were stronger among females and socioeconomically advantaged or healthier‐behavior subgroups. AL mediated up to 35.3% of the birthweight–aging association, whereas CRP showed minimal mediation. Higher birthweight and breastfeeding, particularly in combination, produce the most favorable profiles of multi‐organ biological aging in adulthood, with AL partially mediating these effects. These findings provide novel evidence that prenatal and postnatal nutrition leave enduring, system‐wide imprints on adult aging trajectories.

## Graphical abstract

Higher birthweight was associated with lower biological age gaps across multiple adult organs, whereas breastfeeding showed organ‐specific associations. Joint exposure to higher birthweight and breastfeeding was associated with favorable aging profiles, with allostatic load partially mediating birthweight associations, linking early‐life factors to differences in adult organ aging.

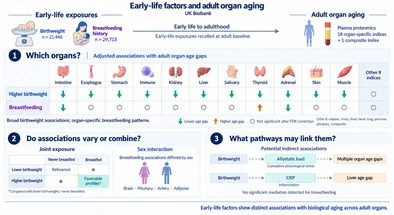

## 1 Introduction

Population aging has become one of the defining demographic transitions of the 21st century, accompanied by a growing burden of chronic diseases and functional decline worldwide (Kroemer et al. 2025). Central to this process is organ‐specific aging, a multidimensional phenomenon reflecting structural and functional deterioration across multiple physiological systems (Tian et al. 2023). Recent advances in imaging and omics have enabled quantitative assessment of aging across organs, revealing remarkable inter‐individual heterogeneity in the rate and pattern of organ decline (Oh et al. 2023; Prattichizzo et al. 2024). Understanding what drives this variability, and identifying modifiable determinants across the life course, has therefore emerged as a key scientific and public health priority.

A substantial body of evidence supports the Developmental Origins of Health and Disease (DOHaD) hypothesis, which posits that early‐life exposures, particularly during the perinatal period, exert lasting influences on organ development and subsequent disease susceptibility (Vickers 2022). Perinatal nutritional factors such as birth weight and breastfeeding capture complementary dimensions of the early‐life nutrients. Birth weight reflects fetal growth and intrauterine conditions, whereas breastfeeding represents postnatal nutritional and immunological support. Low birthweight has been associated with an elevated risk of cardiometabolic disorders (Long et al. 2025) and cognitive impairment (Isaksson et al. 2023) in adulthood, whereas breastfeeding has been linked to improved immune function and metabolic profiles (Burugupalli et al. 2025). These observations suggest that the biological embedding of early‐life conditions may extend beyond individual organs to shape a person's systemic aging trajectory. However, little is known about how perinatal nutritional exposures influence the coordinated aging of multiple organ systems in adulthood.

The mechanisms linking perinatal nutritional factors to organ aging remain poorly understood. The influence of early‐life growth and nutritional conditions on later physiological aging may be modified by subsequent health behaviors and socioeconomic environments, which shape the extent to which early exposures translate into lasting biological effects (Raffington et al. 2021). At the biological level, chronic inflammation and cumulative physiological stress may represent a convergent pathway through which early‐life conditions are biologically embedded (Hinz et al. 2025). Inflammatory markers such as C‐reactive protein (CRP) and composite indices of allostatic load (AL)—reflecting the cumulative burden of metabolic, immune, and neuroendocrine dysregulation—have been recognized as key mediators of biological aging (Feng et al. 2025; Hamlat et al. 2023). Elevated CRP and higher AL scores have been consistently associated with accelerated aging across multiple organ systems (Mazhar et al. 2025). Given that perinatal nutritional exposures shape immune maturation and stress regulation, inflammation and allostatic load likely serve as key intermediaries linking early‐life conditions to organ‐specific aging. However, integrative evidence testing these pathways within a life‐course framework remains scarce in large population cohorts.

In this study, we systematically examined how two key perinatal nutritional exposures, namely birthweight and breastfeeding, relate to organ aging across 19 aging indices in adulthood. Using data from the UK Biobank, a large and deeply phenotyped population cohort, we first quantified the direct associations between perinatal factors and organ‐specific aging indices and then assessed potential modification by sociodemographic and behavioral factors. We further tested whether systemic inflammation and cumulative physiological stress mediate these associations, thereby delineating biological pathways through which early‐life conditions become embedded in later‐life physiology. By integrating developmental, behavioral, and biological perspectives within a population‐scale framework, this study provides a comprehensive model linking perinatal nutritional environments to the trajectory of adult organ aging.

## 2 Methods

### 2.1 Study Population and Data Source

This study was conducted using data from the UK Biobank (UKB), a large, population‐based prospective cohort that recruited approximately 500,000 participants from across the United Kingdom between 2006 and 2010. At enrollment, individuals aged 37–73 years completed comprehensive baseline assessments, including questionnaires on sociodemographic and lifestyle factors, physical and cognitive examinations, and collection of biological samples. These data were subsequently linked to longitudinal electronic health records, enabling integrative analyses across behavioral, biological, and clinical domains. The UK Biobank study was approved by the North West Multi‐Centre Research Ethics Committee (REC reference: 21/NW/0157), and all participants provided written informed consent. Data access for this analysis was obtained under approved UK Biobank Application Number 46478.

For the present study, organ‐specific aging indices constructed in 44,817 participants (Section 2.3) were linked separately to the birthweight and breastfeeding cohorts using participant identifiers. Participants with complete data on the respective exposure, all 19 aging indices, and all covariates included in Model 3 were eligible for analysis, yielding final analytic samples of 21,446 for birthweight and 29,713 for breastfeeding. Baseline characteristics of the two analytic samples are presented in Table 1.

**TABLE 1:** Baseline characteristics of participants included in the birthweight and breastfeeding analyses.

Characteristics	Birthweight (*n* = 21,446)	Breastfeeding (*n* = 29,713)
Age, years (mean (SD))	55.43 (8.21)	55.99 (8.22)
Female sex, *n* (%)	12,910 (60.2)	16,877 (56.8)
White ethnicity, *n* (%)	20,698 (96.5)	27,692 (93.2)
Education (college or higher), *n* (%)	8739 (40.7)	12,323 (41.5)
Townsend Deprivation Index (mean (SD))	−1.44 (3.01)	−1.29 (3.12)
Body size at age 10, *n* (%)		
Thinner	7025 (32.8)	9849 (33.1)
Plumper	3487 (16.3)	4655 (15.7)
About average	10,934 (51.0)	15,209 (51.2)
Birthweight, kg (mean (SD))	3.33 (0.66)	—
Ever breastfed, *n* (%)	—	21,687 (73.0)
Body mass index, kg/m^2^ (mean (SD))	27.21 (4.74)	27.25 (4.69)
Ever smoked, *n* (%)	9274 (43.2)	13,159 (44.3)
Ever drank alcohol, *n* (%)	20,705 (96.5)	28,405 (95.6)
Physical activity, MET‐min/week (mean (SD))	47.50 (59.55)	48.29 (61.33)
Hypertension, *n* (%)	6019 (28.1)	8824 (29.7)
Diabetes, *n* (%)	1559 (7.3)	2450 (8.2)
Coronary heart disease, *n* (%)	2146 (10.0)	3234 (10.9)
Organ‐specific aging indices, mean (SD)		
Adipose	0.04 (0.97)	0.00 (0.98)
Adrenal	−0.03 (0.99)	−0.02 (0.99)
Artery	0.02 (0.98)	−0.01 (0.99)
Brain	−0.04 (0.96)	−0.03 (0.98)
Esophagus	−0.04 (0.98)	−0.01 (1.00)
Heart	0.01 (0.97)	0.00 (0.98)
Immune	−0.04 (0.97)	−0.02 (0.99)
Intestine	−0.05 (0.98)	−0.04 (0.99)
Kidney	−0.05 (0.95)	−0.04 (0.97)
Liver	0.00 (0.99)	−0.02 (0.99)
Lung	−0.04 (0.97)	−0.04 (0.98)
Muscle	−0.03 (0.97)	−0.02 (0.98)
Pancreas	−0.05 (0.96)	−0.03 (0.98)
Pituitary	0.03 (0.99)	0.01 (0.99)
Salivary	−0.02 (0.99)	−0.02 (0.99)
Skin	0.00 (0.99)	−0.02 (1.00)
Stomach	−0.03 (0.96)	−0.02 (0.98)
Thyroid	−0.04 (0.98)	−0.02 (0.99)
Conventional	−0.01 (0.98)	−0.02 (0.99)

*Note:* Characteristics are shown for participants included in the birthweight and breastfeeding analyses, respectively. Continuous variables are shown as mean (SD) and categorical variables as *n* (%). Organ‐specific indices represent *z*‐scored residuals from linear regressions of predicted biological age on chronological age within each organ system.

### 2.2 Definition of Perinatal Nutritional Factors

Two key perinatal nutritional exposures were examined in this study: birthweight and breastfeeding status. Both variables were derived from early‐life information collected at the UK Biobank baseline assessment.

Birthweight was obtained from Data‐Field 20,022 (“Birth weight”), which captures self‐reported birthweight recorded during verbal interviews. Participants were asked to provide their birthweight either in kilograms or in imperial units (pounds and ounces). Birthweight was analyzed as a continuous measure (in kilograms) to preserve variability and maximize statistical power. Implausible values (< 0.5 kg or > 6 kg) were excluded prior to analysis.

Breastfeeding status was derived from Data‐Field 1677 (“Breastfed as a baby”), assessed via a touchscreen questionnaire at baseline. Participants were asked, “Were you breastfed when you were a baby?” Response options included “Yes,” “No,” and “Do not know.” For analysis, participants who responded “Do not know” or had missing data were excluded, and breastfeeding status was coded dichotomously (yes vs. no).

### 2.3 Assessment of Organ‐Specific Aging

Organ‐specific aging indices were derived using plasma proteomic data from the UK Biobank Pharma Proteomics Project (UKB‐PPP). The UKB‐PPP measured 2923 plasma proteins using the Olink Explore 3072 platform in samples collected at baseline from 52,999 participants. After post‐UKB‐PPP quality control, 8182 samples with more than 1000 missing protein values and seven proteins missing in over 10% of samples were excluded, resulting in a final dataset comprising 44,817 participants and 2916 proteins. Missing proteomic values were imputed following the established UKB‐PPP pipeline. Briefly, data were split into training and testing subsets based on assessment centers, protein abundances were standardized using training‐set statistics, and missing values were imputed using a k‐nearest neighbors approach with the number of neighbors defined as the square root of the training sample size (*k* = 152). Model performance was optimized based on the mean absolute error (MAE) of predicted versus chronological age. Detailed procedures have been described previously (Goeminne et al. 2025).

Proteome‐based aging models were then developed using elastic net regression, with chronological age as the outcome and plasma protein profiles as predictors. Model training and evaluation were performed using five‐fold cross‐validation, and performance was assessed based on the mean absolute error between predicted and chronological age. Organ‐specific aging indices were derived from these models as described previously (Oh et al. 2025). For each participant, the organ‐specific age gap was defined as the residual from a linear regression of predicted biological age on chronological age within each organ system. Positive residuals indicate accelerated organ aging, whereas negative residuals reflect decelerated aging. To ensure comparability across organ systems, age gaps were z‐scored within each model. In total, 19 aging indices were generated, including 18 organ‐specific indices representing distinct physiological domains and one composite aging index. These indices have been validated to capture organ‐level deviations from normative aging trajectories (Goeminne et al. 2025; Oh et al. 2025).

### 2.4 Assessment of Covariates

Covariates included age (continuous, years), sex (male or female), ethnicity (White or non‐White), comparative body size at age 10, body mass index (BMI), Townsend Deprivation Index (TDI), educational attainment, smoking, alcohol use, physical activity, hypertension, diabetes, and coronary heart disease. Comparative body size at age 10 was retrospectively self‐reported through the baseline touchscreen questionnaire (Field 1687) and modeled as a three‐category variable: thinner, plumper, or about average. Responses of “Do not know” or “Prefer not to answer” were treated as missing and excluded. BMI (kg/m^2^) was calculated from height and weight measured at baseline. TDI was modeled continuously, with higher scores indicating greater deprivation, and educational attainment was modeled categorically. Smoking and alcohol use were each categorized as ever or never. Physical activity was assessed using self‐reported activity data. Hypertension, diabetes, and coronary heart disease were identified from hospital inpatient ICD‐10 diagnoses (Field 41270) using codes I10–I15, E10–E14, and I20–I25, respectively, and modeled as binary variables (yes or no). The diabetes definition included Type 1, Type 2, and other specified or unspecified forms of diabetes.

### 2.5 Statistical Analyses

Multivariable linear regression models were applied to quantify associations between birthweight and breastfeeding and each of the 19 organ‐specific aging indices. Birthweight and breastfeeding were analyzed separately across three sequential models: Model 1 adjusted for age, sex, and ethnicity; Model 2 additionally included body size at age 10, BMI, TDI, educational attainment, smoking (ever/never), alcohol use (ever/never), and physical activity; and Model 3 further adjusted for hypertension, diabetes, and coronary heart disease. Multicollinearity was assessed using variance inflation factors (all < 5). Potential effect modification by behavioral and socioeconomic characteristics was evaluated via interaction terms, followed by stratified analyses when significant.

Sensitivity analyses included: (1) additional adjustment for medication use (drugs for cholesterol, blood pressure, diabetes, or exogenous hormone therapy), (2) mutual adjustment between birthweight and breastfeeding, and (3) exploration of nonlinearity in birthweight associations using restricted cubic splines.

To assess the combined influence of perinatal nutritional factors, a four‐level joint exposure variable was constructed by dichotomizing birthweight at the median (low vs. high) and cross‐classifying with breastfeeding (ever vs. never), with low birthweight and never breastfed as the reference.

Causal mediation analyses were conducted to investigate whether systemic inflammation (CRP) and multisystem physiological dysregulation (AL score) mediated the observed associations. AL was calculated as a composite of 10 biomarkers across metabolic, cardiovascular, and inflammatory systems, dichotomized into high‐ versus low‐risk strata and summed (range: 0–10) (Castagne et al. 2018). Mediation models adjusted for the same covariates as Model 3, and effects were estimated using nonparametric bootstrapping (1000 simulations), reporting average causal mediation effect (ACME), average direct effect (ADE), and proportion mediated.

All analyses were conducted in R version 4.4.1. Two‐sided *p* < 0.05 was considered statistically significant, with false discovery rate (FDR) correction applied for multiple testing across organ systems.

## 3 Results

### 3.1 Associations Between Perinatal Nutritional Factors and Organ‐Specific Aging

In fully adjusted models (Model 3), birthweight showed consistent inverse associations with organ‐specific biological aging across 11 systems (Figure 1a). Specifically, greater birthweight was linked to lower organ‐specific age gaps in the intestine (*β* = −0.069, 95% CI: −0.089 to −0.050), esophagus (*β* = −0.055, 95% CI: −0.075 to −0.035), stomach (*β* = −0.053, 95% CI: −0.072 to −0.034), immune system (*β* = −0.045, 95% CI: −0.064 to −0.026), kidney (*β* = −0.044, 95% CI: −0.062 to −0.025), liver (*β* = −0.042, 95% CI: −0.062 to −0.022), salivary (*β* = −0.034, 95% CI: −0.054 to −0.014), thyroid (*β* = −0.033, 95% CI: −0.053 to −0.014), adrenal (*β* = −0.029, 95% CI: −0.048 to −0.010), skin (*β* = −0.029, 95% CI: −0.049 to −0.009), and muscle (*β* = −0.027, 95% CI: −0.047 to −0.008). Breastfeeding exposure was associated with decelerated aging in the intestine (*β* = −0.047, 95% CI: −0.071 to −0.022) and adrenal (*β* = −0.030, 95% CI: −0.055 to −0.006), but with accelerated aging in the thyroid (*β* = 0.033, 95% CI: 0.008–0.058). A detailed summary of regression coefficients, 95% confidence intervals, and false discovery rate (FDR)‐adjusted *p*‐values for all perinatal–organ associations is provided in Tables S1 and S2.

**FIGURE 1:**
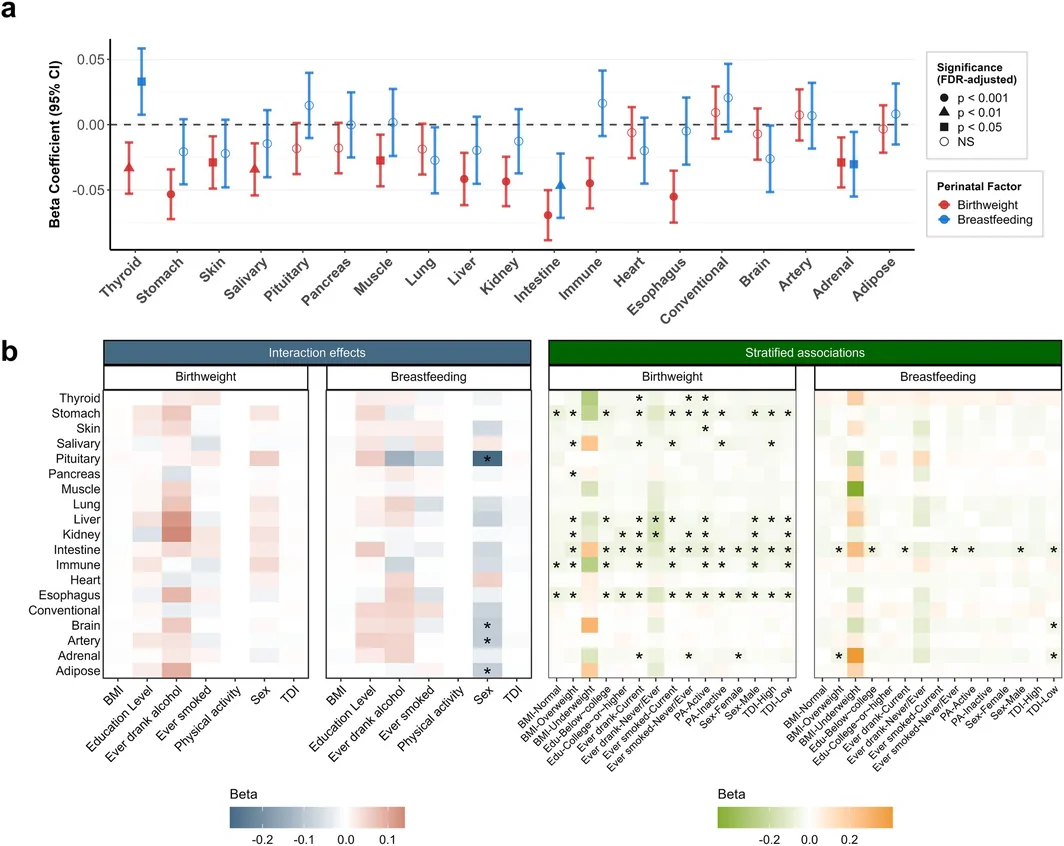
Associations between perinatal factors and organ‐specific biological aging and the modifying roles of behavioral and socioeconomic factors. (a) Fully adjusted associations of birthweight and breastfeeding with 19 organ‐specific biological aging indices. Point shapes reflect FDR‐adjusted statistical significance. (b) Heatmaps displaying interaction effects (left panel) and stratified associations (right panel) for birthweight and breastfeeding across key behavioral and socioeconomic factors, including BMI, smoking, alcohol use, physical activity, educational attainment, sex, and the Townsend Deprivation Index (TDI). Asterisks indicate FDR‐adjusted significance.

Sensitivity analyses supported the robustness of the primary findings. First, additional adjustment for medication use yielded effect estimates that were virtually unchanged, with all previously significant associations persisting. Second, mutual adjustment for the two perinatal exposures largely replicated the main results: the birthweight–organ aging associations remained significant for 9 out of 11 organ systems, and the inverse association between breastfeeding and intestinal aging remained robust. Although the associations between breastfeeding and adrenal and thyroid aging were attenuated, effect directions were consistent with the main analyses, suggesting interdependence between perinatal factors rather than spurious findings (Tables S3 and S4). Third, restricted cubic spline analyses suggested evidence of nonlinearity for some organ‐specific aging indices; however, visual inspection indicated that the overall associations were approximately linear, supporting the use of birthweight as a continuous variable in the main analyses (Figure S1).

### 3.2 Effect Modification by Behavioral and Socioeconomic Factors

As shown in Figure 1b, interaction analyses suggested that breastfeeding exerted stronger protective effects in females, particularly for brain, pituitary, arterial, and adipose aging. Stratified analyses demonstrated two population subgroups in which perinatal advantages were most prominent: individuals with higher BMI and those with lower TDI (indicating more favorable socioeconomic circumstances).

### 3.3 Joint Exposure Analysis

Compared with the reference group, individuals with both high birthweight and breastfeeding demonstrated the most favorable aging profiles across multiple organ systems (Figure 2). Significant reductions in organ‐specific aging indices were observed for the intestine (*β* = −0.13, 95% CI –0.17 to −0.09), kidney (*β* = −0.08, 95% CI –0.12 to −0.04), liver (*β* = −0.07, 95% CI –0.11 to −0.03), adrenal gland (*β* = −0.07, 95% CI –0.11 to −0.03), lung (*β* = −0.06, 95% CI –0.10 to −0.02), and stomach (*β* = −0.09, 95% CI –0.13 to −0.05), suggesting a robust and systemic protective effect associated with this dual exposure.

**FIGURE 2:**
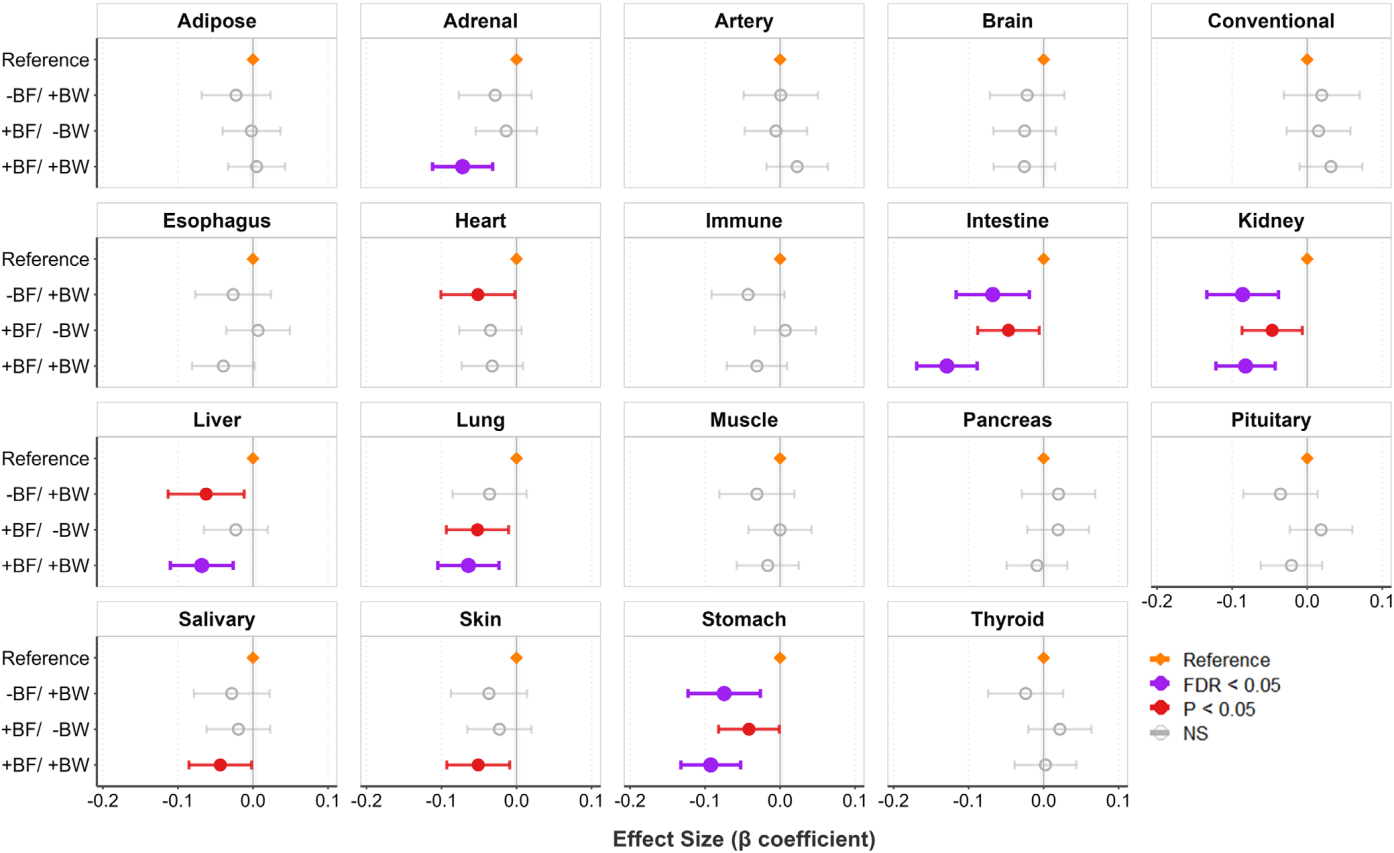
Joint effects of birthweight and breastfeeding on organ‐specific aging. Participants were categorized into four exposure groups based on birthweight (high vs. low) and breastfeeding status (yes vs. no): “Low BW and never BF” (low birthweight, never breastfed), “High BW and never BF” (high birthweight, never breastfed), “Low BW and BF” (low birthweight, breastfed), and “High BW and BF” (high birthweight, breastfed). Regression coefficients (β) and 95% confidence intervals (CIs) were estimated from fully adjusted linear models (Model 3), accounting for age, sex, ethnicity, body size at age 10, BMI, Townsend Deprivation Index, educational attainment, smoking, alcohol use, physical activity, hypertension, diabetes, and coronary heart disease.

Notably, higher birthweight without breastfeeding was also associated with lower aging indices in the kidney, intestine, liver, and stomach, although the estimated associations were generally smaller in magnitude than those observed in the joint exposure group. This suggests indicating a potential additive or synergistic interaction between prenatal and postnatal nutritional advantages. In contrast, the combination of low birthweight and breastfeeding conferred only modest benefits in the kidney, intestine, lung, and stomach, indicating that breastfeeding may partially mitigate, but not fully compensate for, the adverse effects of low fetal growth.

### 3.4 Mediation Analysis: Roles of Inflammation and Allostatic Load

AL emerged as a key mediator between birthweight and aging across multiple organ systems, particularly in stress‐sensitive endocrine and epithelial tissues (Table 2). The strongest indirect effects were identified for skin, where 35.3% of the birthweight benefit was attributable to lower cumulative stress burden. Substantial mediation proportions were also observed for adrenal aging (30.5%) and thyroid aging (19.8%). Relatively moderate contributions were observed for salivary gland (18.1%), kidney (15.2%), and intestine (11.6%), while more modest mediation was noted for esophagus (8.3%), immune aging (7.3%), and stomach (5.0%). Systemic inflammation, indexed by CRP, showed a smaller yet significant pathway restricted to liver aging, mediating 4.8% of the total effect. No significant mediation was detected for breastfeeding with either mediator across any aging outcome.

**TABLE 2:** Mediation effects of allostatic load and inflammation on the association between birthweight and organ‐specific biological aging.

Mediator	Outcome	ACME (95% CI)	ACME *p*	ADE (95% CI)	ADE *p*	Total effect (95% CI)	Total *p*	% mediated
Allostatic load	Adrenal	−0.0086 (−0.0116, −0.0060)	< 0.001	−0.0197 (−0.0420, 0.0012)	0.064	−0.0283 (−0.0510, −0.0081)	0.008	30.5
	Esophagus	−0.0049 (−0.0069, −0.0032)	< 0.001	−0.0538 (−0.0769, −0.0316)	< 0.001	−0.0587 (−0.0817, −0.0362)	< 0.001	8.3
	Immune	−0.0033 (−0.0050, −0.0018)	< 0.001	−0.0413 (−0.0643, −0.0182)	< 0.001	−0.0445 (−0.0675, −0.0219)	< 0.001	7.3
	Intestine	−0.0077 (−0.0101, −0.0053)	< 0.001	−0.0585 (−0.0809, −0.0336)	< 0.001	−0.0662 (−0.0886, −0.0416)	< 0.001	11.6
	Kidney	−0.0077 (−0.0104, −0.0053)	< 0.001	−0.0429 (−0.0662, −0.0176)	< 0.001	−0.0505 (−0.0740, −0.0262)	< 0.001	15.2
	Salivary gland	−0.0059 (−0.0082, −0.0040)	< 0.001	−0.0267 (−0.0514, −0.0043)	0.022	−0.0327 (−0.0573, −0.0101)	0.004	18.1
	Skin	−0.0066 (−0.0088, −0.0046)	< 0.001	−0.0121 (−0.0354, 0.0101)	0.29	−0.0187 (−0.0411, 0.0041)	0.118	35.3
	Stomach	−0.0028 (−0.0045, −0.0015)	< 0.001	−0.0538 (−0.0756, −0.0306)	< 0.001	−0.0566 (−0.0781, −0.0335)	< 0.001	5.0
	Thyroid	−0.0067 (−0.0094, −0.0045)	< 0.001	−0.0272 (−0.0499, −0.0028)	0.022	−0.0339 (−0.0564, −0.0103)	0.006	19.8
CRP levels	Liver	−0.0019 (−0.0034, −0.0007)	< 0.001	−0.0389 (−0.0578, −0.0191)	0.002	−0.0408 (−0.0596, −0.0212)	< 0.001	4.8

*Note:* Average causal mediation effects (ACME) quantify the indirect effects of birthweight on organ‐specific biological aging through allostatic load or circulating C‐reactive protein (CRP) levels. Average direct effects (ADE) reflect the residual effects of birthweight not explained by the mediator. The proportion mediated (%) was calculated as ACME divided by the total effect. Models were adjusted for age, sex, ethnicity, body size at age 10, body mass index (BMI), Townsend Deprivation Index (TDI), educational attainment, smoking, alcohol use, physical activity, hypertension, diabetes, and coronary heart disease.

## 4 Discussion

In this large population‐based study, we mapped multi‐organ biological aging in relation to early‐life nutrition using plasma proteomics. Higher birthweight was broadly associated with slower aging across multiple organs, whereas breastfeeding showed more organ‐specific associations. Individuals with both higher birthweight and breastfeeding exhibited the most favorable aging profiles, highlighting the lasting influence of prenatal and postnatal nutrition on adult aging trajectories beyond traditional disease endpoints.

Birthweight was associated with biological aging across 11 organs, supporting a systemic rather than organ‐isolated pattern and underscoring its role as an integrated marker of early developmental conditions. Accumulating evidence across multiple organ systems provides biological plausibility for these findings. Low birthweight has been linked to impaired intestinal vascular development, reduced villus height, and decreased digestive enzyme activity, suggesting long‐lasting compromises in gut structure and absorptive capacity that may manifest as accelerated intestinal aging later in life (Zou et al. 2023). In parallel, lower birthweight and small‐for‐gestational‐age status have been associated with increased hepatic fat accumulation, whereas higher birthweight appears protective against metabolic dysfunction–associated steatotic liver disease (Ebrahimi et al. 2024). Similar developmental constraints have been described in the kidney, where reduced nephron endowment is associated with lower filtration capacity and increased risk of chronic kidney disease (Lillas et al. 2021), and in the musculoskeletal system, where low birthweight predicts reduced muscle mass, impaired function, and greater susceptibility to sarcopenia (Celik et al. 2024). Collectively, these observations are consistent with developmental programming processes whereby birthweight reflects early‐life conditions shaping long‐term organ integrity through coordinated influences on metabolism (Cohen et al. 2022), vascular maintenance (Zou et al. 2023), gut–microbiota interactions (Ding et al. 2024), and systemic inflammatory burden (Raisi‐Estabragh et al. 2023).

Breastfeeding exhibited heterogeneous, organ‐specific associations with biological aging, indicating that its long‐term effects may depend on distinct developmental pathways. The protective association with intestinal aging is biologically plausible, as gut aging is characterized by epithelial renewal failure, microbiota dysbiosis, increased permeability, and low‐grade systemic inflammation (Wang et al. 2025). Bioactive components of breast milk, including human milk oligosaccharides and immune factors such as secretory IgA, promote beneficial microbial colonization, preserve barrier integrity, and suppress inflammation (Wells et al. 2022), thereby potentially attenuating age‐related gut deterioration. The inverse association with adrenal aging may reflect long‐term modulation of the hypothalamic–pituitary–adrenal axis by breastfeeding (Henshaw 2023). In contrast, the positive association with thyroid aging may reflect compensatory metabolic stress. Variability in iodine and thyroid hormone content of breast milk could increase metabolic load on the developing thyroid, potentially accelerating follicular exhaustion under imbalance (Andersson and Braegger 2022). This interpretation remains speculative, particularly given the complexity of thyroid regulation. Notably, these associations were more evident among individuals with more favorable socioeconomic or metabolic profiles, indicating that the long‐term impact of breastfeeding on organ aging may be contingent on later‐life environmental context.

Individuals with both higher birthweight and breastfeeding demonstrated the most favorable multi‐organ aging profiles, consistent with complementary prenatal and postnatal influences. Birthweight may reflect intrauterine growth conditions that establish organ‐level structural and metabolic reserve, while breastfeeding may modulate postnatal immune maturation and stress regulation (Ebrahimi et al. 2024; Querter et al. 2022; Salem et al. 2022; Yin et al. 2024). The convergence of these processes likely contributes to enhanced organ resilience and slower biological aging across systems.

Our mediation analyses provide empirical support for a shared systemic pathway linking early growth to multi‐organ aging, demonstrating that AL and CRP partially mediate the association between birthweight and biological aging across multiple organ systems. AL emerged as a central pathway, with marked organ‐specific heterogeneity. This pattern aligns with life‐course frameworks suggesting that early growth conditions shape long‐term stress regulatory capacity, thereby influencing cumulative physiological wear and tear in adulthood (Etzel et al. 2023). The strongest mediation was observed in stress‐sensitive endocrine and epithelial tissues, including skin, adrenal, and thyroid, which are tightly integrated within neuroendocrine–immune networks and exhibit heightened responsiveness to chronic stress exposure (Hamilton et al. 2024; Li 2023; Zhang et al. 2024). Relatively moderate mediation in organs such as the kidney and intestine suggests that stress‐related dysregulation constitutes one of multiple contributing pathways, whereas weaker mediation in the esophagus, stomach, and immune aging may indicate a greater dependence on local regenerative processes or tissue‐specific microenvironmental regulation that are not fully captured by systemic AL (Hanson and Gluckman 2014). This contrast underscores the added value of AL as an integrative construct capturing multisystem stress‐related dysregulation beyond isolated inflammatory processes. The absence of detectable mediation for breastfeeding suggests that its long‐term effects may operate through earlier developmental or localized biological pathways rather than chronic stress accumulation (Lee et al. 2023).

Interaction and stratified analyses underscore a broader bio–social integration perspective, whereby the long‐term effects of perinatal nutritional factors on organ‐specific aging are shaped jointly by early biological endowment and later‐life social and behavioral contexts. Associations of birthweight and breastfeeding with organ aging differed across socioeconomic status, health behaviors, and sex. This pattern suggests that early developmental trajectories do not operate in isolation but are shaped by cumulative exposures across the life course. In particular, the more pronounced protective effects of higher birthweight observed among individuals with higher educational attainment, lower deprivation, and non‐smoking status are consistent with the cumulative advantage framework in life‐course epidemiology (Ben‐Shlomo and Kuh 2002). Similarly, stronger protective associations of breastfeeding among women suggest potential sex‐specific modulation, possibly reflecting interactions between early nutrition, lifelong hormonal milieu, and gender‐patterned health behaviors (Sandovici et al. 2022).

Several limitations warrant consideration. The observational and predominantly cross‐sectional design limits causal inference. Perinatal nutritional exposures, including birth weight and breastfeeding, were based on self‐reported data and may be subject to recall or reporting bias, although such misclassification would likely attenuate observed associations toward the null. Breastfeeding was assessed as a binary exposure, precluding evaluation of duration, exclusivity, or dose–response relationships. In addition, proteomics‐based aging indices provide scalable proxies of organ‐specific aging but may not fully capture structural or functional aging assessed by imaging or histopathology. Future studies incorporating prospectively collected perinatal data, more granular breastfeeding measures, and longitudinal assessments of biological aging are needed to further clarify the causal pathway.

In summary, this large population‐based analysis suggests that early‐life nutritional factors, particularly birthweight and breastfeeding, are associated with long‐term patterns of biological aging across multiple organ systems. Higher birthweight was linked to broadly favorable aging profiles, breastfeeding showed more organ‐specific associations, and their joint exposure conferred the most consistent protection. Mediation analyses further implicate systemic stress and inflammatory regulation as partial pathways linking early development to adult aging. Together, these findings underscore the importance of a life‐course perspective and highlight early‐life nutrition as a meaningful contributor to heterogeneity in biological aging trajectories.

## Author Contributions

W.W. and X.Q. contributed to material preparation, data collection, and data analysis. W.W. drafted the first version of the manuscript. B.C., B.Z., D.H., J.H., S.C., X.Y., and J.F. contributed to the preparation of figures and tables. C.P., Y.G., L.L., Y.W., H.L., and Y.J. contributed to the literature review. F.Z. contributed to the study design. All authors critically reviewed the manuscript and approved the final version.

## Funding

This work was supported by the Shaanxi Provincial Health Research Innovation Capacity Improvement Program (Grant No. 2026TD‐08) and the Fundamental Research Funds for the Central Universities of Xi’an Jiaotong University (Grant No. xzy022025090).

## Disclosure

Transparency Declaration: As the lead author and guarantor of this manuscript, I hereby affirm that this manuscript is an honest, accurate, and transparent account of the study being reported; that no important aspects of the study have been omitted.

## Ethics Statement

UK Biobank has ethical approval from the North West Multi‐Centre Research Ethics Committee (REC reference: 21/NW/0157). All participants provided written informed consent. Access to the UK Biobank data used in this study was obtained under approved application number 46478.

## Conflicts of Interest

The authors declare no conflicts of interest.

## Supporting information


**Table S1:** Associations between birthweight and organ‐specific biological aging across models.
**Table S2:** Associations between breastfeeding and organ‐specific biological aging across models.
**Table S3:** Sensitivity analyses of the associations between birthweight and organ‐specific biological aging indices.
**Table S4:** Sensitivity analyses of the associations between breastfeeding and organ‐specific biological aging indices.
**Figure S1:** Nonlinear associations between birthweight and organ‐specific biological aging indices.

## Data Availability

The data that support the findings of this study are available from the UK Biobank upon application. Restrictions apply to the availability of these data, which were used under license for the current study and are therefore not publicly available. The analysis code used in this study is available from the corresponding author upon reasonable request.
