# Supplementary material for: Birthweight and Breastfeeding Shape Patterns of Biological Aging Across Adult Organs
Source: Aging Cell. 2026 Oct 9;25(10):e70762. doi: 10.1111/acel.70762 (PMC13650733; doi:10.1111/acel.70762)
Supplement: Supplementary file 1 [file ACEL-25-e70762-s001.docx]

Supplementary Materials for:

**Birthweight and Breastfeeding Shape Patterns of Biological Aging Across Adult Organs**

**Supplementary Table S1.** Associations Between Birthweight and Organ-specific Biological Aging Across Models

**Supplementary Table S2.** Associations Between Breastfeeding and Organ-specific Biological Aging Across Models

**Supplementary Table S3.** Sensitivity Analyses of the Associations Between Birthweight and Organ-specific Biological Aging Indices

**Supplementary Table S4.** Sensitivity Analyses of the Associations Between Breastfeeding and Organ-specific Biological Aging Indices

**Supplementary Figure S1.** Nonlinear Associations Between Birthweight and Organ-Specific Biological Aging Indices

**Table S1.** **Associations Between Birthweight and Organ-specific Biological Aging Across Models**

| Organ system | Model 1 |  |  | Model 2 |  |  | Model 3 |  |
| --- | --- | --- | --- | --- | --- | --- | --- | --- |
|  | *β* (95% CI) | FDR *P* |  | *β* (95% CI) | FDR *P* |  | *β* (95% CI) | FDR *P* |
| Adipose | –0.000 (–0.018, 0.017) | 0.98 |  | –0.004 (–0.022, 0.014) | 0.743 |  | –0.003 (–0.021, 0.015) | 0.76 |
| Adrenal | **–0.035 (–0.053, –0.016)** | 0.001 |  | **–0.034 (–0.053, –0.014)** | 0.002 |  | **–0.029 (–0.048, –0.010)** | 0.012 |
| Artery | 0.007 (–0.011, 0.025) | 0.505 |  | 0.006 (–0.014, 0.025) | 0.67 |  | 0.007 (–0.012, 0.027) | 0.577 |
| Brain | –0.019 (–0.038, –0.001) | 0.059 |  | –0.014 (–0.034, 0.006) | 0.257 |  | –0.007 (–0.027, 0.012) | 0.579 |
| Esophagus | **–0.060 (–0.078, –0.041)** | <0.001 |  | **–0.056 (–0.075, –0.036)** | <0.001 |  | **–0.055 (–0.075, –0.035)** | <0.001 |
| Heart | –0.014 (–0.032, 0.005) | 0.175 |  | –0.011 (–0.030, 0.009) | 0.373 |  | –0.006 (–0.026, 0.013) | 0.621 |
| Immune | **–0.043 (–0.061, –0.025)** | <0.001 |  | **–0.051 (–0.070, –0.031)** | <0.001 |  | **–0.045 (–0.064, –0.026)** | <0.001 |
| Intestine | **–0.080 (–0.099, –0.062)** | <0.001 |  | **–0.076 (–0.096, –0.057)** | <0.001 |  | **–0.069 (–0.089, –0.050)** | <0.001 |
| Kidney | **–0.059 (–0.077, –0.041)** | <0.001 |  | **–0.056 (–0.075, –0.037)** | <0.001 |  | **–0.044 (–0.062, –0.025)** | <0.001 |
| Liver | **–0.037 (–0.056, –0.018)** | <0.001 |  | **–0.045 (–0.065, –0.025)** | <0.001 |  | **–0.042 (–0.062, –0.022)** | <0.001 |
| Lung | **–0.022 (–0.040, –0.004)** | 0.028 |  | –0.020 (–0.039, 0.000) | 0.102 |  | –0.019 (–0.038, 0.001) | 0.133 |
| Muscle | **–0.021 (–0.040, –0.003)** | 0.037 |  | **–0.031 (–0.051, –0.011)** | 0.007 |  | **–0.027 (–0.047, –0.008)** | 0.021 |
| Pancreas | **–0.031 (–0.049, –0.013)** | 0.002 |  | **–0.025 (–0.044, –0.005)** | 0.033 |  | –0.018 (–0.037, 0.001) | 0.137 |
| Pituitary | **–0.026 (–0.045, –0.008)** | 0.008 |  | –0.018 (–0.038, 0.001) | 0.117 |  | –0.018 (–0.038, 0.001) | 0.137 |
| Salivary | **–0.035 (–0.053, –0.016)** | 0.001 |  | **–0.036 (–0.056, –0.016)** | 0.002 |  | **–0.034 (–0.054, –0.014)** | 0.004 |
| Skin | **–0.031 (–0.049, –0.012)** | 0.002 |  | **–0.032 (–0.052, –0.012)** | 0.006 |  | **–0.029 (–0.049, –0.009)** | 0.016 |
| Stomach | **–0.065 (–0.083, –0.047)** | <0.001 |  | **–0.061 (–0.080, –0.042)** | <0.001 |  | **–0.053 (–0.072, –0.034)** | <0.001 |
| Thyroid | **–0.036 (–0.054, –0.017)** | <0.001 |  | **–0.035 (–0.054, –0.015)** | 0.002 |  | **–0.033 (–0.053, –0.014)** | 0.004 |
| Conventional | 0.012 (–0.006, 0.031) | 0.229 |  | 0.004 (–0.015, 0.024) | 0.743 |  | 0.009 (–0.011, 0.029) | 0.477 |

***Notes:*** Model 1 adjusted for age, sex, and ethnicity. Model 2 additionally adjusted for body size at age 10, BMI, Townsend Deprivation Index, educational attainment, smoking, alcohol use, and physical activity. Model 3 further adjusted for hypertension, diabetes, and coronary heart disease. FDR *P* indicates false discovery rate–corrected p values. Boldface indicates associations with FDR-corrected *P* < 0.05.

**Table S2. Associations Between Breastfeeding and Organ-specific Biological Aging Across Models**

| Organ system | Model 1 |  |  | Model 2 |  |  | Model 3 |  |
| --- | --- | --- | --- | --- | --- | --- | --- | --- |
|  | *β* (95% CI) | FDR *P* |  | *β* (95% CI) | FDR *P* |  | *β* (95% CI) | FDR *P* |
| Adipose | **-0.044 (-0.067, -0.022)** | <0.001 |  | 0.008 (-0.016, 0.031) | 0.637 |  | 0.008 (-0.015, 0.032) | 0.586 |
| Adrenal | **-0.046 (-0.070, -0.021)** | <0.001 |  | **-0.031 (-0.056, -0.007)** | 0.033 |  | **-0.030 (-0.055, -0.006)** | 0.044 |
| Artery | -0.018 (-0.041, 0.006) | 0.175 |  | 0.005 (-0.020, 0.031) | 0.743 |  | 0.007 (-0.018, 0.032) | 0.663 |
| Brain | **-0.037 (-0.061, -0.012)** | 0.006 |  | -0.030 (-0.055, -0.004) | 0.053 |  | -0.026 (-0.052, -0.001) | 0.106 |
| Esophagus | **0.042 (0.018, 0.066)** | 0.002 |  | -0.005 (-0.030, 0.021) | 0.765 |  | -0.005 (-0.031, 0.021) | 0.760 |
| Heart | -0.018 (-0.042, 0.006) | 0.175 |  | -0.025 (-0.050, 0.000) | 0.105 |  | -0.020 (-0.045, 0.005) | 0.201 |
| Immune | -0.006 (-0.029, 0.018) | 0.648 |  | 0.013 (-0.012, 0.039) | 0.373 |  | 0.016 (-0.009, 0.041) | 0.305 |
| Intestine | **-0.074 (-0.098, -0.051)** | <0.001 |  | **-0.050 (-0.075, -0.025)** | <0.001 |  | **-0.047 (-0.071, -0.022)** | 0.001 |
| Kidney | **-0.030 (-0.054, -0.006)** | 0.021 |  | -0.017 (-0.042, 0.008) | 0.270 |  | -0.013 (-0.037, 0.012) | 0.422 |
| Liver | **-0.053 (-0.077, -0.028)** | <0.001 |  | -0.022 (-0.048, 0.004) | 0.155 |  | -0.020 (-0.045, 0.006) | 0.213 |
| Lung | **-0.059 (-0.083, -0.035)** | <0.001 |  | -0.029 (-0.055, -0.004) | 0.053 |  | -0.027 (-0.053, -0.002) | 0.087 |
| Muscle | 0.009 (-0.015, 0.033) | 0.505 |  | 0.000 (-0.025, 0.026) | 0.972 |  | 0.002 (-0.024, 0.027) | 0.922 |
| Pancreas | **-0.034 (-0.057, -0.010)** | 0.010 |  | -0.003 (-0.028, 0.022) | 0.835 |  | 0.000 (-0.025, 0.025) | 0.989 |
| Pituitary | 0.024 (0.001, 0.047) | 0.061 |  | 0.016 (-0.009, 0.041) | 0.307 |  | 0.015 (-0.010, 0.040) | 0.362 |
| Salivary | 0.006 (-0.018, 0.031) | 0.641 |  | -0.015 (-0.041, 0.011) | 0.338 |  | -0.015 (-0.040, 0.011) | 0.375 |
| Skin | **-0.059 (-0.084, -0.035)** | <0.001 |  | -0.024 (-0.050, 0.002) | 0.117 |  | -0.022 (-0.048, 0.004) | 0.178 |
| Stomach | -0.021 (-0.044, 0.003) | 0.123 |  | -0.024 (-0.049, 0.001) | 0.116 |  | -0.021 (-0.046, 0.004) | 0.186 |
| Thyroid | **0.044 (0.020, 0.069)** | <0.001 |  | **0.032 (0.007, 0.058)** | 0.033 |  | **0.033 (0.008, 0.058)** | 0.032 |
| Conventional | 0.007 (-0.017, 0.031) | 0.624 |  | 0.018 (-0.008, 0.044) | 0.257 |  | 0.021 (-0.005, 0.047) | 0.201 |

***Notes:*** Model 1 adjusted for age, sex, and ethnicity. Model 2 additionally adjusted for body size at age 10, BMI, Townsend Deprivation Index, educational attainment, smoking, alcohol use, and physical activity. Model 3 further adjusted for hypertension, diabetes, and coronary heart disease. FDR *P* indicates false discovery rate–corrected p values. Boldface indicates associations with FDR-corrected *P* < 0.05.

**Table S3. Sensitivity Analyses of the Associations Between Birthweight and Organ-specific Biological Aging Indices**

| Organ system | Sensitivity 1 |  |  | Sensitivity 2 |  |
| --- | --- | --- | --- | --- | --- |
|  | *β* (95% CI) | FDR *P* |  | *β* (95% CI) | FDR *P* |
| Adipose | -0.003 (-0.022, 0.015) | 0.777 |  | -0.010 (-0.030, 0.009) | 0.469 |
| Adrenal | **-0.028 (-0.048, -0.009)** | 0.014 |  | -0.024 (-0.045, -0.003) | 0.074 |
| Artery | 0.007 (-0.013, 0.026) | 0.588 |  | 0.014 (-0.007, 0.035) | 0.341 |
| Brain | -0.008 (-0.027, 0.012) | 0.569 |  | -0.008 (-0.029, 0.013) | 0.543 |
| Esophagus | **-0.055 (-0.075, -0.035)** | <0.001 |  | **-0.064 (-0.085, -0.042)** | <0.001 |
| Heart | -0.007 (-0.027, 0.012) | 0.585 |  | -0.007 (-0.028, 0.014) | 0.591 |
| Immune | **-0.044 (-0.064, -0.025)** | <0.001 |  | **-0.042 (-0.063, -0.021)** | <0.001 |
| Intestine | **-0.069 (-0.088, -0.050)** | <0.001 |  | **-0.072 (-0.093, -0.051)** | <0.001 |
| Kidney | **-0.042 (-0.061, -0.024)** | <0.001 |  | **-0.046 (-0.067, -0.026)** | <0.001 |
| Liver | **-0.042 (-0.062, -0.022)** | <0.001 |  | **-0.045 (-0.067, -0.024)** | <0.001 |
| Lung | -0.018 (-0.038, 0.001) | 0.136 |  | -0.016 (-0.037, 0.005) | 0.269 |
| Muscle | **-0.027 (-0.047, -0.008)** | 0.021 |  | **-0.028 (-0.050, -0.007)** | 0.035 |
| Pancreas | -0.018 (-0.037, 0.002) | 0.142 |  | -0.017 (-0.038, 0.003) | 0.215 |
| Pituitary | -0.019 (-0.039, 0.000) | 0.120 |  | -0.023 (-0.044, -0.001) | 0.108 |
| Salivary | **-0.034 (-0.054, -0.014)** | 0.004 |  | **-0.032 (-0.054, -0.011)** | 0.014 |
| Skin | **-0.029 (-0.049, -0.009)** | 0.017 |  | -0.022 (-0.044, 0.000) | 0.122 |
| Stomach | **-0.052 (-0.072, -0.033)** | <0.001 |  | **-0.058 (-0.079, -0.038)** | <0.001 |
| Thyroid | **-0.033 (-0.053, -0.014)** | 0.004 |  | **-0.038 (-0.059, -0.016)** | 0.003 |
| Conventional | 0.009 (-0.011, 0.029) | 0.475 |  | 0.017 (-0.005, 0.038) | 0.266 |

***Notes:*** Two complementary sensitivity analyses were conducted to evaluate the robustness of the associations between birthweight and organ-specific aging indices. Sensitivity Analysis 1: Regression models were built on Model 3 and additionally adjusted for medication use, including drugs for cholesterol, blood pressure, diabetes, or exogenous hormone therapy. Sensitivity Analysis 2: To address potential interdependence between perinatal exposures, regression models included mutual adjustment for breastfeeding. Boldface indicates associations with FDR-corrected *P* < 0.05.

**Table S4. Sensitivity Analyses of the Associations Between Breastfeeding and Organ-specific Biological Aging Indices**

| Organ system | Sensitivity 1 |  |  | Sensitivity 2 |  |
| --- | --- | --- | --- | --- | --- |
|  | *β* (95% CI) | FDR *P* |  | *β* (95% CI) | FDR *P* |
| Adipose | 0.007 (-0.016, 0.031) | 0.629 |  | 0.013 (-0.015, 0.042) | 0.483 |
| Adrenal | **-0.032 (-0.056, -0.007)** | 0.037 |  | -0.029 (-0.058, 0.001) | 0.147 |
| Artery | 0.006 (-0.019, 0.032) | 0.690 |  | 0.007 (-0.023, 0.038) | 0.665 |
| Brain | -0.026 (-0.052, -0.001) | 0.102 |  | -0.014 (-0.045, 0.016) | 0.483 |
| Esophagus | -0.004 (-0.030, 0.021) | 0.788 |  | 0.002 (-0.028, 0.033) | 0.877 |
| Heart | -0.020 (-0.045, 0.005) | 0.199 |  | -0.009 (-0.039, 0.021) | 0.624 |
| Immune | 0.016 (-0.009, 0.041) | 0.322 |  | 0.012 (-0.018, 0.042) | 0.532 |
| Intestine | **-0.048 (-0.072, -0.023)** | <0.001 |  | **-0.050 (-0.080, -0.020)** | 0.005 |
| Kidney | -0.013 (-0.037, 0.012) | 0.431 |  | -0.020 (-0.049, 0.009) | 0.334 |
| Liver | -0.019 (-0.045, 0.006) | 0.225 |  | -0.012 (-0.043, 0.019) | 0.532 |
| Lung | -0.028 (-0.053, -0.003) | 0.074 |  | **-0.040 (-0.070, -0.010)** | 0.035 |
| Muscle | 0.002 (-0.024, 0.027) | 0.929 |  | 0.009 (-0.021, 0.040) | 0.624 |
| Pancreas | 0.000 (-0.025, 0.025) | 0.987 |  | -0.003 (-0.033, 0.027) | 0.877 |
| Pituitary | 0.014 (-0.011, 0.039) | 0.397 |  | 0.017 (-0.013, 0.047) | 0.437 |
| Salivary | -0.015 (-0.041, 0.010) | 0.355 |  | -0.015 (-0.046, 0.016) | 0.483 |
| Skin | -0.024 (-0.049, 0.002) | 0.142 |  | -0.018 (-0.049, 0.013) | 0.422 |
| Stomach | -0.021 (-0.046, 0.004) | 0.169 |  | -0.027 (-0.056, 0.003) | 0.177 |
| Thyroid | **0.032 (0.006, 0.057)** | 0.038 |  | 0.027 (-0.003, 0.058) | 0.177 |
| Conventional | 0.021 (-0.005, 0.047) | 0.199 |  | 0.013 (-0.018, 0.044) | 0.532 |

***Notes:*** Two complementary sensitivity analyses were conducted to evaluate the robustness of the associations between birthweight and organ-specific aging indices. Sensitivity Analysis 1: Regression models were built on Model 3 and additionally adjusted for medication use, including drugs for cholesterol, blood pressure, diabetes, or exogenous hormone therapy. Sensitivity Analysis 2: To address potential interdependence between perinatal exposures, regression models included mutual adjustment for birthweight. Boldface indicates associations with FDR-corrected *P* < 0.05.

**Figure S1.** **Nonlinear Associations Between Birthweight and Organ-Specific Biological Aging Indices**

***Notes:*** Restricted cubic spline models were applied to evaluate potential nonlinearity in the relationships between birthweight and 19 organ-specific biological aging indices. Only associations with overall significance (P < 0.05) are visualized. Shaded bands represent 95% confidence intervals, and representative birthweight values are highlighted to illustrate the magnitude of deviation across the distribution. Across organ systems, spline curves indicated minimal departure from linearity, suggesting that birthweight-related differences in aging risk were largely monotonic.
